# Phenotypic and genotypic characterization of Colombian clinical isolates of *Sporothrix* spp.

**DOI:** 10.7705/biomedica.6898

**Published:** 2023-08-31

**Authors:** Laura C. Álvarez-Acevedo, María C. Zuleta-González, Óscar M. Gómez-Guzmán, Álvaro L. Rúa-Giraldo, Orville Hernández-Ruiz, Juan G. McEwen-Ochoa, Martha E. Urán-Jiménez, Myrtha Arango-Arteaga, Rosely M. Zancopé-Oliveira, Manoel Marques Evangelista de Oliveira, María del P. Jiménez-Alzate

**Affiliations:** 1 Grupo de Micología Médica, Departamento de Microbiología y Parasitología, Facultad de Medicina, Universidad de Antioquia, Medellín, Colombia Universidad de Antioquia Universidad de Antioquia Medellín Colombia; 2 Posgrado de Biología, Instituto de Biología, Facultad de Ciencias Exactas y Naturales, Universidad de Antioquia, Medellín, Colombia Universidad de Antioquia Universidad de Antioquia Medellín Colombia; 3 Grupo de Biología Celular y Molecular, Corporación para Investigaciones Biológicas, Universidad de Antioquia, Medellín, Colombia Universidad de Antioquia Universidad de Antioquia Medellín Colombia; 4 Escuela de Microbiología, Universidad de Antioquia, Medellín, Colombia Universidad de Antioquia Universidad de Antioquia Medellín Colombia; 5 Laboratorio de Micología, Instituto Nacional de Infectología Evandro Chagas, Fundação Oswaldo Cruz, Rio de Janeiro, RJ, Brazil Fundação Oswaldo Cruz Fundação Oswaldo Cruz Rio de Janeiro Brazil

**Keywords:** Sporothrix, sporotrichosis, phenotype, genotype, *Sporothrix* spp., esporotricosis, fenotipo, genotipo

## Abstract

**Introduction.:**

For over a century, *Sporothrix* schenckii was considered the sole species responsible for sporotrichosis. In 2007, scientific community confirmed the disease could be caused by various *Sporothrix* species. These species differed in their virulence factors and their antifungal sensitivity.

**Objective.:**

This study aims to characterize 42 Colombian clinical isolates of *Sporothrix* spp. phenotypically and genotypically.

**Material and methods.:**

Forty-two clinical isolates were characterized using phenotypic methods. It involved various culture media to determine their growth range at different temperatures and to assess the type and distribution of pigment and colony texture. Microscopic morphology was evaluated through microcultures, as well as the conidia diameter, type of sporulation, and morphology. Additionally, the assimilation of carbohydrates was selected as a physiological trait for species identification. Genotyping of 40 isolates was performed through partial amplification of the calmodulin gene, followed by sequence analysis.

**Results.:**

Molecular studies enabled the identification of 32 isolates of *S. schenckii* and 8 isolates of *S. globosa*. The combination of phenotypic and genotypic methods eased these species characterizations and the recognition keys development based on parameters such as growth diameter at 25 and 30 °C, colony texture (membranous or velvety) on potato dextrose agar, and microscopic morphology with predominance of pigmented triangular, elongated oval globose, or subglobose conidia.

**Conclusions.:**

Confirmation of the phenotypic characteristics and molecular analysis is crucial for identifying *Sporothrix* species and determining adequate treatment. This study represents the first phenotypical and genotypical characterization of clinical isolates of *Sporothrix* spp. reported in Colombia.

## Resumen

**Introducción.:**

Por más de un siglo se creyó que *Sporothrix* schenckii era la única especie responsable de la esporotricosis. Sin embargo, en el 2007, se consideró que podría ser causada por diferentes especies de *Sporothrix*, que difieren en sus factores de virulencia y su sensibilidad a los antifúngicos.

**Objetivo.:**

Caracterizar fenotípica y genotípicamente 42 aislamientos clínicos colombianos de *Sporothrix* spp.

**Materiales y métodos.:**

Se caracterizaron 42 aislamientos clínicos mediante métodos fenotípicos. Se usaron varios medios de cultivo para determinar el rango de crecimiento a diferentes temperaturas, el tipo y la distribución del pigmento, y la textura de las colonias. Se evaluó la morfología microscópica por microcultivos mediante la determinación del diámetro, el tipo de esporulación y la morfología de las conidias. La asimilación de carbohidratos se usó como una característica fisiológica para identificar las especies. La genotipificación de los 40 aislamientos se llevó a cabo mediante la amplificación parcial del gen que codifica para la calmodulina y se confirmó por secuenciación.

**Resultados.:**

Mediante estudios moleculares, se identificaron 32 aislamientos de *S. schenckii* y ocho de *S. globosa*. La combinación de métodos fenotípicos y genotípicos permitió caracterizar las especies y construir claves para su reconocimiento, con base en parámetros como el diámetro de crecimiento a 25 y 30 °C, la textura de las colonias (membranosa, aterciopelada) en agar papa dextrosa y la morfología microscópica con predominio de conidias (triangulares pigmentadas, ovales globosas elongadas, subglobosas).

**Conclusiones.:**

La caracterización fenotípica y los análisis moleculares son necesarios para identificar las especies de *Sporothrix* y, de esta forma, elegir el tratamiento indicado. Esta es la primera caracterización fenotípica y genotípica reportada de aislamientos clínicos colombianos de *Sporothrix* spp.

Sporotrichosis is a subcutaneous granulomatous mycosis with a subacute or chronic evolution course that afflicts humans and mammals worldwide and produces different clinical manifestations. The disorder is produced by dimorphic fungi included in the clinical clade of *Sporothrix* spp. ^1^.

In Latin America, sporotrichosis is the most prevalent subcutaneous mycosis. It regularly exhibits changes in its epidemiological patterns. Classically, sporotrichosis is acquired by traumatic inoculation of the fungus through contaminated materials, such as plants, thorns, or soil, penetrating the host’s subcutaneous tissues. Nonetheless, zoonotic transmission by domestic cats has been observed, thus changing the accepted transmission paradigm of this disease ^2^.

For over a century, *Sporothrix schenckii* was considered the only species responsible for all cases of sporotrichosis ^3^^,^^4^. However, in 2007, Marimon *et al*. ^5^ proposed to split *S. schenckii* into several molecular siblings by phylogenetic analysis of the calmodulin gene (exons 3-5). *Sporothrix* is a diverse *genus* with wide genetic and ecological diversity, reflected in the many different associations between organisms and their hosts or niches ^6^.

Currently, *Sporothrix* comprises 53 species divided into clinical and environmental clades. The clinical clade includes four species causing human and animal infections: *S. brasiliensis*, *S. schenckii*, *S. globosa*, and *S. luriei*. Most *Sporothrix* species belong to the environmental clade: *S. pallida* complex, *S. stenoceras* complex, *S. candida* complex, *S. inflata* complex, and *S. gossypina* complex; and feature lower pathogenic potential toward mammals. Environmental *Sporothrix* species are usually associated with decaying wood, plant debris, soil, insects, etc. However, some species of this clade have been reported as the causative agent of sporothricosis cases: *S. pallida* complex (*S. chilensis*, *S. humicola*, *S. mexicana*, *S. pallida*), and *S. stenoceras* complex ^6^^,^^7^.

Identification and differentiation of clinical clades species is important due to the variability of their virulence and geographic distribution. Additionally, their sensitivity to antifungals reveals different levels of resistance. These variations are also noticed in the frequency of the species causing the mycosis.

Sporotrichosis is the most common subcutaneous mycosis in Colombia, with a prevalence rate of 0.1 to 0.5% in one report ^8^ or eight cases per 100,000 patients in another ^9^. In Colombia, the epidemiology and microbiology information about the causative agents of sporotrichosis is unknown since the related studies were conducted in big cities, such as Medellin and Cali, and were focused merely on the disease epidemiology and clinical manifestations ^10^^,^^11^. In the Medical Mycology Laboratory at the *Facultad de Medicina* of the *Universidad de Antioquia* (*Medellín*), where we collected the present data, *S. schenckii* was considered the only causative agent of sporotrichosis, but the characteristics of the isolates were not explored. This prompted us to undertake a detailed phenotypical and genotypical characterization of those clinical isolates.

## Materials and methods

### Isolates

We characterized 42 isolates previously identified as *Sporothrix* spp. These isolates were preserved according to the following protocol.

### Subcultures

The isolates were sub-cultured on Mycosel agar and incubated at room temperature for one month. Once we confirmed the isolates’ growth, some colonies were transferred to tubes containing distilled water for short-term storage at room temperature, and others, mineral oil and skim milk medium for -20°C storage, for further studies.

### Phenotypic characterization

*Macroscopic morphology*. The growth range, pigmentation, and colony texture were assessed in 42 isolates. An inoculum of 5 x 10^5^ conidia/ml in 10 μl was plated on potato dextrose agar and corn meal agar (CMA) per triplicate. The plates were incubated at 25, 30 and 37 °C. The growth diameter was measured in millimeters, longitudinally and transversely, using a ruler every 7, 14, 21, 28, and 40 days. During the same period, we evaluated qualitatively pigments, morphology, and texture of the grown colonies. All isolates were cultured in dark incubators.

*Microscopic morphology*. We evaluated the conidia produced by the mycelial form using microcultures. After ten days of incubation at 25 °C, we estimated morphology, diameter, and conidiation type in potato dextrose agar and corn meal agar. Ten photographs were taken for each isolate, with a 100fold magnification, using a Leica DM 500™ camera adapted to a Leica ICC 50W™ microscope. We employed the SE Micrometrics Premium 4 ACCU- SCOPE software to measure the area and the perimeter of 100 conidia (100 hyaline and 100 dematiaceous or pigmented) per isolate. This information was compiled in an Excel™ database for further statistical analysis.

*Transition from mycelial to yeast form*. Two enriched culture media induced the yeast morphology in each isolate: Brain-heart infusion (BHI, BBLTM; ref. 211065. Franklin Lakes, NJ, USA) supplemented with 2% glucose (Sigma, ref. G5400-250G) (BHI-I); and supplemented with 0.1% L-cysteine (Sigma, ref. C-7755. St. Louis, MO, USA), 5% sheep blood, and 2% glucosa (BHI-II). These media were incubated at 37°C with 5% CO_2_.

The complete transition from mycelium to yeast form was determined by conducting multiple cultures every eight days, until creamy colonies obtention. Confirmation was achieved through microscopic observation of blue lactophenol-stained cotton corresponding to the colonies, revealing oval structures analogous to blastoconidia and hyphae absence.

*Carbohydrate assimilation*. Carbohydrate assimilation was evaluated in all isolates in the yeast form using API AUX 20 (Biomerieux®, Marcy-l’Etoile- France) after confirmation of the complete transition from mycelium to yeast form, as mentioned. The protocol was modified by adding 200 μl of yeast suspension and reading at 8-10 days after seeding the wells. Turbid wells were considered positive compared to the negative control, lacking a source of carbohydrates. We evaluated the following carbohydrates: glucose, glycine, α-ketoglutarate, arabinose, xylose, adonitol, xylitol, galactose, inositol, sorbitol, methyl D-glucosidase, N-acetylglucosamine, cellobiose, lactose, maltose, sucrose, trehalose, melezitose and raffinose.

### Genotypic characterization

DNA was extracted and purified from the yeast phase of all 40 isolates after three days of growth using the protocol recommended by Norgen Biotek Corporation for fungi/yeast genomic DNA (Thorold, ON, Canada). For the remaining two isolates, DNA was extracted from the mycelial phase using the phenol-chloroform and isoamyl alcohol method. The DNA was quantified using the NanoDrop™ One/OneC Microvolume UV-Vis spectrophotometer. A expected protein ratio (260/280) within the range of 1.8-2.0 was needed to ensure the absence of protein contamination.

Partial amplification of the calmodulin gene (*CALM1*) was performed from genomic DNA using conventional PCR with primers described by O'Donnell *et al*. ^12^: CL1 (5’-GAR TWC AAG GAG GCC TTC TC-3 ‘) and CL2A (5’ -TTT TTG CAT CAT GAG TTG GAC-3 ‘). These primers generate an 800 pb amplicon corresponding to exons 3 and 5 of this gene.

### Phylogenetic analysis

The products were sent to Macrogen Inc. (Geumcheon-gu, Seoul, South Korea) for sequencing using the ABI 3730XL DNA sequencing technology with the quality criterion of QV20. The obtained sequences were edited using the Geneious, version 11.0.2, software. Partial calmodulin gene sequences from *Sporothrix* spp. isolates, reported in the GenBank, were also included to improve phylogenetic tree support. The IqTree, version 1.4.4, software was used to determine the evolutionary model and the relationships between the isolates. A maximum likelihood-based phylogenetic tree was built using the Kimura two-parameter models and Jukes Cantor with a 1,000 bootstrap.

## Results

### Phenotypic characterization

**Figure 1:**
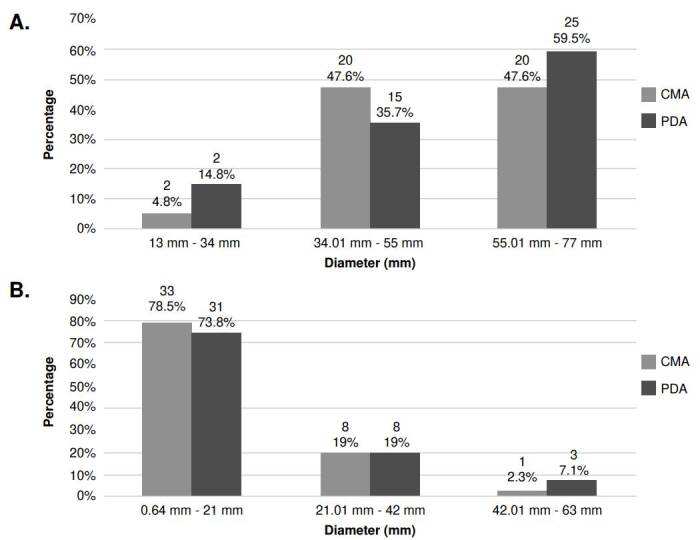
Growth range of growth of *Sporothrix* spp. clinical isolates in their mycelial phase. **A.** Comparison of growth diameter in potato dextrose agar (PDA) and corn meal gar (CMA) at 25 °C and after 40 days of observation. The number of isolates (n) are located at the top level of the numbers in percentages for each bar. **B.** Comparison of PDA and CMA growth diameter at 30°C at 40 days of observation. The number of isolates (n) are located at the top of each bar.

*Growth ranges*. The isolates’ growth was measured every seven days for a 40 day-period. The isolates showed similar growth ranges in the evaluated culture media (potato dextrose agar and corn meal agar). However, their growth differed at the tested temperatures, with colonies growing more at 25 °C than at 30 °C (figure 1A and 1B). Growth was not observed in none of the 28 isolates at 37 °C, regardless of the culture media used (data not shown). Among the 14 isolates that exhibited growth at 37 °C, the range was between 0.3 and 2.5 mm in potato dextrose agar, and between 0.2 and 1.9 mm in corn meal agar.

### Colony textures of Sporothrix spp. clinical isolates

Macroscopic characteristics among the isolates were better defined in potato dextrose agar at 25 °C, while in the corn meal agar, all had a similar texture, not allowing differentiation among the isolates. Therefore, observations of the colony texture and pigment were made only in potato dextrose agar.

**Figure 2:**
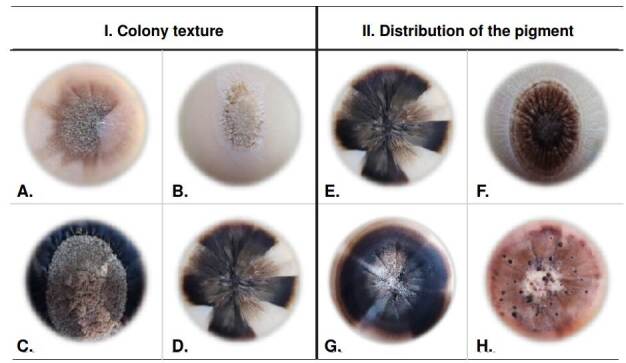
Phenotypic characteristics of *Sporothrix* spp. clinical isolates colonies. I. Texture of the *Sporothrix* spp. clinical isolates colonies after 40 days of observation: Velvety (A), central hairy (B), membranous (C), and cerebriform (D). II. Pigment distribution of the *Sporothrix* spp. clinical isolates colonies after 40 days of observation: Spotted heterogeneous (A), centralized heterogeneous (B), sectorized heterogeneous (C), and dotted heterogeneous (D).

The texture of *Sporothrix* spp. clinical isolates exhibited a heterogeneous range of colonies. We found the membranous aspect prevailed among 50% of the isolates (n=21), but this trait was also present in 42,85% of others (n=37), sharing other textures such as velvety, central hairy, and cerebriform-like. A single texture, different from the membranous, was less frequent: centralhairy texture colonies corresponded to 2.38% (n=1) and with velvety texture to 2.38% (n=1). The cerebriform aspect shared textures with those previously described in 11.9% (n=5), but it was not observed as a separate texture. Figure 2-I illustrates previously described textures, and table 1 shows the frequencies of the observed different textures.

**Table 1:** Texture of *Sporothrix* spp. isolates colonies in their mycelial phase after 40 days of culture in potato dextrose agar at 25 °C (N=42)

Texture	%	n
Membranous	50	21
Membranous and central hairy	19.05	8
Membranous and velvety	9.52	4
Membranous and cerebriform	2.38	1
Membranous, velvety and cerebriform	2.38	1
Membranous, central hairy and velvety	2.38	1
Membranous, central hairy, cerebriform and velvety	2.38	1
Central hairy	4.76	2
Velvety	2.38	1
Central hairy and velvety	2.38	1

### Pigments produced by Sporothrix spp. clinical isolates at the mycelial phase

The isolates in their mycelial phase presented various pigment types after 40 days of growth in potato dextrose agar at 25 °C, even within the same isolate. The pigments observed included light types (beige, light brown) and dark types (dark brown, black). The pigments were associated with the size of the colonies: Smaller-sized isolates (22-35 mm) tended to develop dark pigments; medium-size (35.1-58 mm) and biggersize colonies (58.1-76mm) did not show a tendency toward any pigment tone and exhibited pigments ranging from light to dark tonalities.

Distribution of pigment in the mycelial colonies was registered as follows: Homogeneous 33% (n=14), spotted heterogenous 33% (n=14), sectorized heterogenous 26% (n=11), centralized heterogenous 5% (n=2), and dotted heterogenous 3% (n=1). We also studied pigments tonality and distribution in the mycelial colonies. Light pigments exhibited homogenous distribution in 19% (n=8), while dark had a homogenous distribution in 14.2% (n=6) of the isolates. Spotted heterogenous distribution was observed in 11.9% (n=5) of the isolates, and sectorized heterogenous distribution in 2.3% (n=1) of the total.

In some isolates (n=22) we observed a mixture of light and dark pigments and, among this group, sectorized heterogenous distribution in 23.8% (n=10) and spotted heterogenous distribution in 21.42% (n=9) of the isolates. Lower frequencies were obtained for centralized heterogenous and dotted heterogenous distributions, occurring in 4.76% (n=2) and 2.38% (n=1) of the isolates, respectively. Examples of colony texture are shown in figure 2-II.

### Sporothrix spp. conidia and their microscopic morphology in clinical isolates

**Figure 3:**
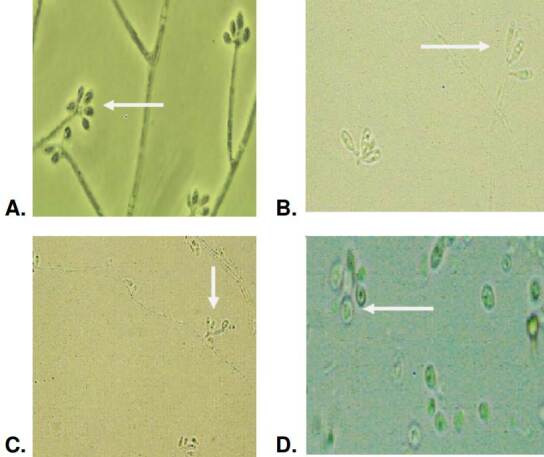
Morphology of hyaline conidia produced by *Sporothrix* spp. clinical isolates and observed at 100-fold magnification: Oval conidia arranged in flower form (A), oval elongated conidia (B) at 40-fold magnification (C), and globose conidia (D).

The observed hyaline conidia had an average area of 4.1 μm^2^ and perimeter of 8.7 pm. The elongated oval shape predominated in this type of hyaline conidia and was present in all isolates (100%). The elongated-oval form was common and identified in 70% of the isolates. The globose and subglobose forms were present in 65% of these conidia (figure 3). Most of the hyaline conidia were organized in a flower shape around a vesicle, a form characteristically described for *Sporothrix* spp. The conidia were produced in a sympodial on denticulate conidiogenous cells.

**Figure 4:**
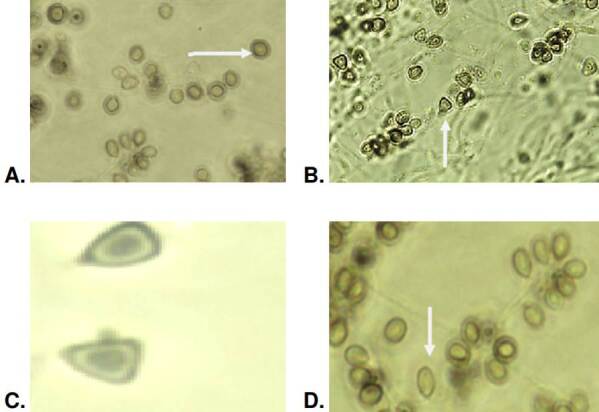
Morphology of dematiaceous conidia produced by *Sporothrix* spp. clinical isolates and observed at 100-fold magnification: Globose and subglobose conidia (A), triangular conidia (B, C), and oval conidia (D).

It was not possible to obtain sporulation in one isolate, so the morphology, as described below, was evaluated only in 41. The dematiaceous or pigmented conidia had an average area of 5.6 μm^2^ and a perimeter of 9.6 μm. In these dematiaceous conidia, the globose and subglobose forms predominated in 27 (65%) isolates out of the evaluated 41. Acropleurogenic sporulation was observed in this conidia form. The presence of oval- dematiaceous conidia organized in a flower-like arrangement was identified in 60% (n=25) of the isolates. In 30% (n=13) of the isolates, we found triangular conidia, primarily organized side by side on the hyphae as sessile acropleurogenic conidia (figure 4).

### Sporothrix spp. clinical isolates: Patterns of carbohydrate assimilation

The evaluation of carbohydrates assimilation, determined by the API 20 test in the 42 isolates, showed that all isolates were capable of assimilating glucose, glycine, xylose, sorbitol, N-acetylglucosamine, maltose, sucrose and trehalose. However, important differences were found in the assimilation of the carbohydrates raffinose (40%), α-ketoglutarate (38%), and inositol (35%).

### Sporothrix spp. clinical isolates: Transition from mycelium to yeast form

The transition from the mycelium to the yeast form was achieved by culturing each isolate four times in fresh culture media, with intervals of seven days. The yeast forms were obtained using two different culture media: BHI-I supplemented with 2% glucose, resulting in the yeast phase for 37 out of 42 isolates (88%), and BHI-II supplemented with 0.1% L-cysteine, 5% blood, and 2% glucose, which allowed all isolates (100%) to change to transit to the yeast form.

### Phylogenetic analysis of Sporothrix spp. clinical isolates

Isolate genotyping was performed by partial amplification of the calmodulin gene. The alignment included 40 clinical isolates from the *Grupo de Micología Médica*, *Departamento de Microbiología y Parasitología*, *Facultad de Medicina*, *Universidad de Antioquia* and 162 sequences from the Genbank database, representing pathogenic *Sporothrix* species such as *S. brasiliensis*, *S. schenckii*, *S. globosa*, *S. mexicana*, and *S. chilensis*, as well as environmental species like *S. brunneviolacea*, *S. pallida S. variecibatus*, and *S. lignivorus*, (used as an outgroup).

**Figure 5:**
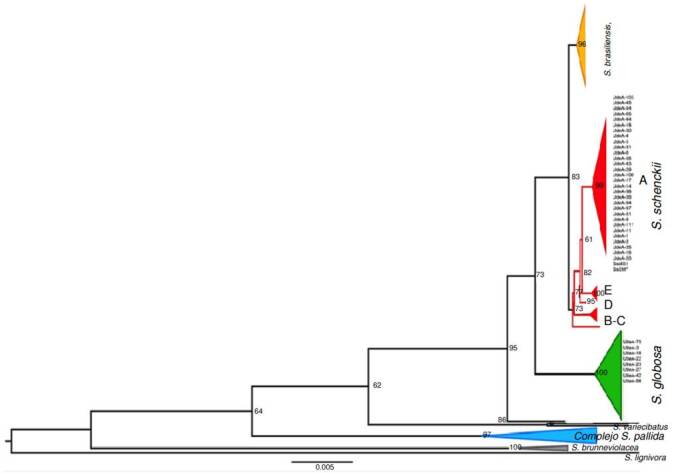
Phylogenetic relationships of the *Sporothrix* spp. clinical isolates. Phylogenetic tree was built from the partial sequences of the calmodulin gene, using the maximum likelihood method. The bootstrap or replicas was 1,000. Branches show the percentages.

Out of the sequenced 42 isolates, 32 were identified as *S. schenckii*, and eight isolates were identified as S. globose (figure 5). Two isolates were identified as *Sarocladium kiliense* by BLAST analysis. However, these sequences were excluded from the alignments as they were determined to be laboratory contaminants. All sequences were analyzed by BLAST and showed identification ranging from 99 to 100%. The sequences were deposited in the Genbank.

### Phenotypic differentiation by species

The molecular identification of the clinical isolates and the differences observed in their phenotypic characteristics, including growth at different temperatures, colony texture and pigment, and conidia microscopic morphology, allowed for the grouping shown in table 2.

**Table 2:** Characterization of *Sporothrix* spp. clinical isolates: Molecular identification and phenotypic findings

Specie	Growth 25°C		Growth 30°C		Growth 37°C	Colonies PDA 25°C	Microscopic morphology
	PDA (mm)	CMA (mm)	PDA (mm)	CMA (mm)	PDA-CMA (mm)		
*S. schenkii*	57.4±8.3	59.63±8.3	18.32±12.3	15.3±2.3	Yes	Membranous	Triangular, oval elongated
*S. globosa*	44.7±11.5	41.3±14.5	9.1±10	7.5±7.9	No	Velvety	Globoses

## Discussion

The main finding of the present study was the successful specieslevel characterization of 40 *Sporothrix* spp. clinical isolates obtained from patients with sporotrichosis in the department of Antioquia, Colombia. Partial sequencing of the calmodulin gene revealed 32 isolates identified as *S. schenckii*, while the remaining eight corresponded to *S. globosa*.

The *S. schenckii* isolates demonstrated a heterogeneous group based on all evaluated phenotypic parameters. We observed that mycelial colonies of *S. schenckii* isolates grown at 25 °C were larger regardless of the culture media used, while at 30 °C, their growth tended to decrease. At 37 °C, the colony size was even smaller in both culture media.

In contrast, *S. globosa* isolates exhibited small mycelial colonies when grown at 25 °C. At 30 °C, in both culture media, the colonies were much smaller than the *S. schenckii* isolates in our study. *Sporothrix globosa* isolates were unable to grow in the nutritional media at 37 °C, consistent with previous phenotypic studies ^5^ reporting their incapacity to grow at this temperature with the employed culture media. Consequently, the transition from mycelial to yeast form at 37 °C was achieved in 100% of the isolates cultured with supplemented BHI-II medium and in 88% of the cases in non-supplemented BHI-I medium.

These findings suggest that supplemented BHI-II medium, also used for the transition of *Histoplasma capsulatum*^13^, could be recommended for studying the yeast form of other dimorphic fungi. In our study, BHI-II medium allowed the yeast form to grow in all tested *Sporothrix* spp. Clinical isolates. In contrast, in 2010, de Oliveira *et al*. ^14^ reported *S. globosa* growth in potato dextrose agar at 37 °C, suggesting significant phenotypic variability likely associated with genetic variability. This variability could indicate varying expression of *S. globosa* phenotypic characteristics according to the geographical origin ^5^^,^^6^.

Overall, our study provides valuable insights into the characterization of *Sporothrix* spp. species at 25 °C, as their growth characteristics had not been previously evaluated at this temperature in other phenotypic studies conducted with this fungus ^5^.

Our data differ from results obtained in other studies incubating the *S. schenckii* complex species at 30 °C in potato dextrose agar and corn meal agar ^5^. In our study, we observed smaller colony sizes at this temperature than those reported by Rodrigues *et al*. in 2013 ^15^ and Camacho *et al*. in 2015 ^16^. They described colony size ranges above 50 mm for *S. schenckii* and greater than 30 mm for *S. globosa* colonies. In 2007, Marimon *et al*. ^5^ reported colony sizes ranging from 33 to 36 mm for *S. schenckii* and from 18 to 40 mm for *S. globosa*, also higher than the sizes observed in our study.

In 2002, another study by Mesa-Arango *et al*. ^17^ analyzed the growth of Colombian isolates from different geographical origins at temperatures of 35 °C and 37 °C. Although these temperatures were different from those evaluated in our study, the findings were similar. The Colombian isolates exhibited lower thermotolerance with a higher percentage of inhibition at these temperatures compared to isolates from other countries (Mexico and Guatemala), which showed statistically significant differences among them. Based on these observations, we can conclude that the growth of Colombian *Sporothrix* spp. isolates is affected at temperatures above 30 °C.

In this study, we evaluated qualitatively the production and distribution of pigments in all isolates. In half of the *S. globosa* isolates (n=4), we detected mixed pigments (light and dark) with heterogeneous distributions, including spotting, centralized, distributed by sectors, and stippled patterns. Conversely, in the remaining *S. globosa* isolates, the distribution was homogeneous, with dark pigments predominance (n=3) compared to the light pigments (n=1).

Among the isolates identified as *S. schenckii*, we observed pigment diversity, including light, mixed, and dark pigments with the mentioned distributions, except for the stippling pattern, found exclusively in *S. globosa* isolates. Previous studies evaluating the phenotypic characteristics of *Sporothrix* spp. have not described the pigment type produced by these fungi. Chemical methods should be implemented to enhance these findings and to identify and quantify produced pigments by *Sporothrix* species.

Regarding pigment assessment, our analysis revealed significant diversity in the colony texture among the evaluated clinical isolates with a predominant membranous aspect. In the isolates identified as *S. globosa*, the dominant texture was velvety-like when grown on potato dextrose agar at 25 °C. Seven of these isolates exhibited this characteristic, along with the cerebriform and central-hairy aspects. Additionally, one of the two isolates that presented a mixture of all four textures, belonged to this species. The isolates identified as *S. schenckii* had a predominant membranous, followed by combinations of central, velvety, and cerebriform hairy textures. Similarly, in *S. globosa* one isolate presented all four textures.

This study does not allow us to specify a characteristic aspect for each species, but similarly to other works, it shows that colonies of the *Sporothrix* species tend to have a predominant membranous texture ^18^. The variation in macroscopic characteristics of the isolates raises suspicion about the possible existence of another species distinct from *S. schenckii*. In the case of the clinical isolates analyzed in this study, our concerns were confirmed with the finding and identification of *S. globosa*. We conducted phenotypic evaluations in potato dextrose agar medium because in corn meal agar all isolates exhibited similar pigment and texture. Cerebriform or centrally hairy colony appearance is not reported because its expression is probably affected by isolates’ multiple in vitro culture passages over the years, causing variability from its original form.

The microscopic morphological findings of this study align with those reported by Marimon *et al*. in 2007 ^5^ and Camacho *et al*. in 2015 ^16^. Conidial morphology descriptions in *S. schenckii* isolates revealed triangularshaped conidia, also known as sessile conidia. All isolates presenting this morphology belong to this species. On the other hand, none of the *S. globosa* isolates formed this type of conidia. They had predominance of globose (round) and sub-globose conidia.

Another significant difference observed in our study is the presence of elongated-to-oval conidia in 28 isolates of *S. schenckii* allocated in IIa clade according to the molecular biology, contrasting with Marimon *et al*. findings: Only two isolates belonging to IIb clade produced that type of conidia ^5^. Regarding the conidia diameter, we found differences between the two species. In *S. schenckii*, the average area and perimeter of dematiaceous and hyaline conidia were higher compared to *S. globosa* conidia from isolates. These findings align with those presented by Marimon *et al*. in 2007 concerning the area, although they did not report the perimeter ^5^. This study has enriched the knowledge of the microscopic aspects of prevalent species within the *Sporothrix* spp. clinical clade, allowing their characterization and differentiation.

Regarding carbohydrate assimilation, the carbohydrates commonly used to differentiate between *Sporothrix* species are raffinose, sucrose, and adonitol or ribitol ^5^. Previous studies reported that *S. globosa* does not assimilate raffinose ^5^, a finding also observed in our study for *S. globosa* and several *S. schenckii* isolates. Therefore, the assimilation of this carbohydrate does not allow for discrimination between the species. We observed differences in the assimilation of aketoglutarate and inositol, but this does not enable the distinction between the two species. The evaluation of carbohydrate assimilation using the Auxonograma API AUX 20 for species of *Sporothrix* spp. has not been reported before. The reason for evaluating the assimilation of these carbohydrates by clinical isolates of *Sporothrix* spp. by this method, was the inability to obtain adonitol by any means.

The *S. schenckii* complex has been divided into species by clades as follows: Clade I, *S. brasiliensis*; clade II, *S. schenckii*; clade III, *S. globose*; clade IV, *S. mexicana*; and clade V, *S. albicans/pallida*^5^. All of our isolates identified as *S. schenckii* were grouped in clade IIa, a subgroup limited to isolates from South American endemic areas, consistent with reports by Mesa-Arango *et al*. in 2002 ^17^, Marimon *et al*. in 2007 ^5^, Zhang *et al*. in 2015 ^18^, Madrid *et al*. in 2009 ^19^, and Rodrigues *et al*. in 2013 ^15^. These reports described the prevalence of *S. globosa* in Colombian isolates, demonstrating the widespread distribution of this species.

The phylogenetic analysis of this study, based on the partial amplification of the calmodulin gene, resulted in a topology tree like those built by the mentioned authors. This similarity demonstrates the importance of utilizing the *CALM1* gene for speciation within the clinical clade of sporotrichosis ^15^^,^^20^^,^^21^. The calmodulin gene has been widely used due to its synonymous substitutions in protein-coding regions, indicating a high degree of conservation and preservation of its original function. However, it contains parsimonious informative sites throughout its sequence, allowing for differentiation between species. This characteristic has led to a large number of available sequences of this gene in public databases and facilitated the development of rapid conventional PCR diagnostic tests using specific primers to identify the causative species of this mycosis ^22^^,^^23^.

The primary objective of this investigation was to phenotypically and genotypically characterize clinical isolates, previously identified as *S. schenckii* and *S. globosa*, to differentiate between the two species.

The results of this study indicate that the Colombian clinical isolates identified as *S. schenckii* exhibit a high degree of phenotypic heterogeneity. However, we could not establish a definitive pattern to differentiate between isolates corresponding to *S. schenckii* and *S. globosa*. Furthermore, Colombian isolates do not possess a higher genetic variability than isolates of *S. schenckii* from other regions of the world. Based on the findings of this research, we concluded that phenotypic characteristics assist in defining the genus and identifying major differences among *Sporothrix* species, but they do not provide a precise species identification. Confirmation of exact species would require the use of molecular biology techniques.
